# The Death at the London Hospital

**Published:** 1893-09-23

**Authors:** 


### THE DEATH AT THE LONDON HOSPITAL.

### INQUEST AND VERDICT.

We take the following account from the *Pall Mall Gazette:* The inquest on the body of little Michael Francis Spallond, the two-year-old son of a waterside labourer, living in Caroline Street, Commercial Road, was held on the 19th inst. by Coroner Baxter at the London Hospital. The child died in a bath, and Mr. Edward Bedford, solicitor, appeared on behalf of its relatives, to assist in discovering the circumstances under which the fatality occurred.

The mother stated that, on the advice of a local doctor, she took her child to the hospital in order that it might undergo an operation. Last Saturday a messenger came from the hospital to fetch her, and when she arrived the doctor told her that there had been a sad accident, and her child was dead. The boy had been placed in a bath, and although the doctor did not say so in as many words, she got the impression that her child had been drowned.

The House Surgeon's Evidence.

Mr. J. F. Johns, house surgeon at the hospital, said the child was brought to the hospital for an operation. That was done, and as the child did not seem to get on well afterwards, he ordered hot hip baths three times a day for periods of twenty minutes to half an hour. At a quarter to twelve he was in the ward, and inquired of the sister how the child was going on. He was told the boy was better, and was in the bath. He left the ward and walked down the corridor to the Central Hall, and almost immediately afterwards he was fetched by Nurse Phelps. He went to the bath-room and found the child dead, and the sister was applying the proper treatment under what she considered to be the circumstances. He measured the depth of the water, and found it just under six inches. Less water than that would have been quite useless for the desired purpose. The bath was in such a position that it could have been seen from any part of the ward—the Princess Beatrice ward. The bath, which was on a table, was a narrow, shallow bath, forty-eight inches long. There had been no accident ever occur in the bath to his knowledge.

The Coroner said that under the circumstances the post-mortem had been made by the highest authority, Dr. Smith, and he would not ask Mr. Johns about that part of the case.

In reply to Mr. Bedford, witness said that the treatment the sister was applying to the child was identical with the treatment for drowning, but was not necessarily treatment for drowning. It was to restore animation from whatever cause animation had been lost.

The Nurse's and Sister's Evidence.

Nurse Phelps said she bathed the child on Saturday morning. She covered its shoulders and placed a board across the bath for it to place its hands upon, in accordance with the usual practice. She was a fully qualified nurse, and had been at the hospital for two years and five months. She was away nearly half an hour. At twenty to twelve she left the child sitting in the bath. She returned at five or ten minutes past twelve and found the child curled up in the water dead. She could not swear whether its mouth was under or above the water. She applied artificial respiration and summoned the sister.

The Coroner: Your absence seems a long one; can you explain it?—I was myself engaged, and the idea of danger never entered my head.

Are you instructed not to leave children when they are being bathed?—Those are the Matron's instructions.

In this case you did not look at it as a bath, there being so small a quantity of water?—No; it was more like an application.

A Juror: Is it usual to leave children in the bath unattended?—Yes, it is usual to leave cases like that unattended. I have never left one so long before, though, as I have taken them in toys, or sweets, or something to drink.

A Juror: It is my opinion that the child was drowned.

The Coroner: You must not determine before hearing the evidence. There is the medical evidence to come presently.

Another Juror: It was sheer neglect.

The Coroner: Do not make the case more painful than it must be.

The Juror: It was painful for the child too.

Sister Lekie said that she instructed Nurse Phelps to bath the child, and to show a probationer how it was done. She went into the bath-room a minute before a quarter to twelve, while Nurse Phelps was away, and the child was then happy and well.

The Coroner: Should a child be left so long in your opinion?—No, sir.

But if it had cried, it could have been heard?—Yes, the door was wide open.

Result of Post-mortem.

Dr. Frederick J. Smith, Lecturer on Forensic Medicine at the London Hospital, said he had made the post-mortem, and had found no congestion of the brain or lungs, no water in the stomach, no blood on either side of the heart, and, in fact, no trace of death from drowning. He frankly admitted that the child did at first appear to have been drowned, and 999 people in a thousand would have come to that conclusion; but as a matter of fact the child had been drowned after death. It must have fallen down into the water after dying from other causes. If he (witness) had been there himself he should have started artificial respiration on the assumption that death was due to drowning, and so would everybody. It was only after fact after fact, each corroborating the other, came to his knowledge that he felt bound to assume that the child was not drowned. Then he searched for some other cause of death, and found that a large stone in the right kidney had dropped into the passage to the bladder, blocking that passage. In his opinion death was due to the shock caused by the shifting of the calculus, probably assisted by the impure condition of the child's blood.

Cross-examined by Mr. Bedford, witness said he had yesterday tried an experiment on a conscious child, and it was most difficult to get it to slip down into the water as he held it.

A verdict of death from natural causes was returned.

